# Nurr1 Represses Tyrosine Hydroxylase Expression via SIRT1 in Human Neural Stem Cells

**DOI:** 10.1371/journal.pone.0071469

**Published:** 2013-08-14

**Authors:** Tae Eun Kim, Ji Sun Seo, Jae Won Yang, Min Woong Kim, Rukhsana Kausar, Eunhye Joe, Bo Yeon Kim, Myung Ae Lee

**Affiliations:** 1 Brain Disease Research Center, Ajou University School of Medicine, Suwon, Korea; 2 Graduate School of Biomedical Sciences, Ajou University School of Medicine, Suwon, Korea; 3 Department of Pharmacology, Ajou University School of Medicine, Suwon, Korea; 4 Chemical Biology Research Center, and World Class Institute, KRIBB, Ochang, Korea; Temple University School of Medicine, United States of America

## Abstract

Nurr1 is an orphan nuclear receptor best known for its essential role in the development and maintenance of midbrain dopaminergic (DA) neurons. During DA neurogenesis, Nurr1 directly targets human tyrosine hydroxylase (hTH). Here we investigated this targeting to identify the molecular mechanisms by which Nurr1 regulates DA neurogenesis. We previously cloned the hTH promoter and found three consensus elements for Nurr1 binding: NBRE-A, -B, and -C. In the present study, gel retardation and luciferase assays using hTH constructs showed that Nurr1 preferentially bound to NBRE-A, through which it mediated transcriptional activity. Furthermore, Nurr1 displayed dual-function transcriptional activities depending on the cell type. In DA-like SH-SY5Y cells, Nurr1 dose-dependently stimulated hTH-3174 promoter activity by 7- to 11-fold. However, in the human neural stem cell (hNSC) line HB1.F3, Nurr1 strongly repressed transcription from the same promoter. This repression was relieved by mutation of only the NBRE-A element and by nicotinamide [an inhibitor of class III histone deacetylases (HDACs), such as SIRT1], but not by trichostatin A (an inhibitor of class I and II HDACs). SIRT1 was strongly expressed in the nucleus of HB1.F3 cells, while it was localized in the cytoplasm in SH-SY5Y cells. ChIP assays of HB1.F3 cells showed that Nurr1 overexpression significantly increased the SIRT1 occupancy of the NBRE-A hTH promoter region, while low SIRT1 levels were observed in control cells. In contrast, no significant SIRT1 recruitment was observed in SH-SY5Y cells. These results indicate that differential SIRT1 localization may be involved in hTH gene regulation. Overall, our findings suggest that Nurr1 exists in dual transcriptional complexes, including co-repressor complexes that can be remodeled to become co-activators and can fine-tune hTH gene transcription during human DA neurogenesis.

## Introduction

The dopaminergic neurons of the midbrain dopaminergic (mdDA) system have been studied extensively in relation to Parkinson’s disease, and many studies have explored the possibility of using cell replacement therapy with stem cells in future treatments [1], [2], [3], [4]. Stem cells could be exploited as an unlimited source of transplantable dopaminergic (DA) neurons. However, in order to engineer stem cells with mdDA characteristics, the appropriate dopaminergic phenotype needs to be obtained through molecular coding [5], [6], [7], [8]. Therefore, much effort has been made to unravel the multi-step process that produces a genuine mdDA neuronal population in vivo, as this is thought to hold the key to successfully engineering stem cells in vitro.

Nurr1 has been shown to be essential for mdDA neuron development because Nurr1-knockout animals lack tyrosine hydroxylase (TH) and other DA characteristics [9], [10]. Nurr1 is required for sustained expression of DA cell-specific genes, normal cell migration, target area innervation, and cell survival [10], [11]. Nurr1 overexpression in stem cells may be important for efforts establishing cell replacement therapies in Parkinson’s disease [12], [13], [14]. Nurr1 may also be associated more directly with neurodegenerative disease because mutations in the human Nurr1 gene have been identified in familial Parkinson’s disease [15]. However, despite intense interest in understanding the development of DA cells, Nurr1 regulation of genes important in DA neuron development has been rarely investigated.

The gene encoding TH, the rate-limiting enzyme in dopamine synthesis, is a well-known target of Nurr1. The TH gene harbors Nurr1 binding elements (NBRE) in its promoter [16], [17], and several reports have shown that Nurr1 regulates the TH gene transcription in cell lines and primary cultures of rodent or human cells [16], [17], [18]. Interestingly, the results were contradictory for the human and rodent models regarding the mechanism underlying TH gene regulation. In rodent cell culture, Nurr1 induces TH expression in both neural precursor and differentiated cells [19], [20], [21], [22]. However, Nurr1 has a minimal impact on human TH gene regulation in human neural precursor cells [17], [23].

**Figure 1:**
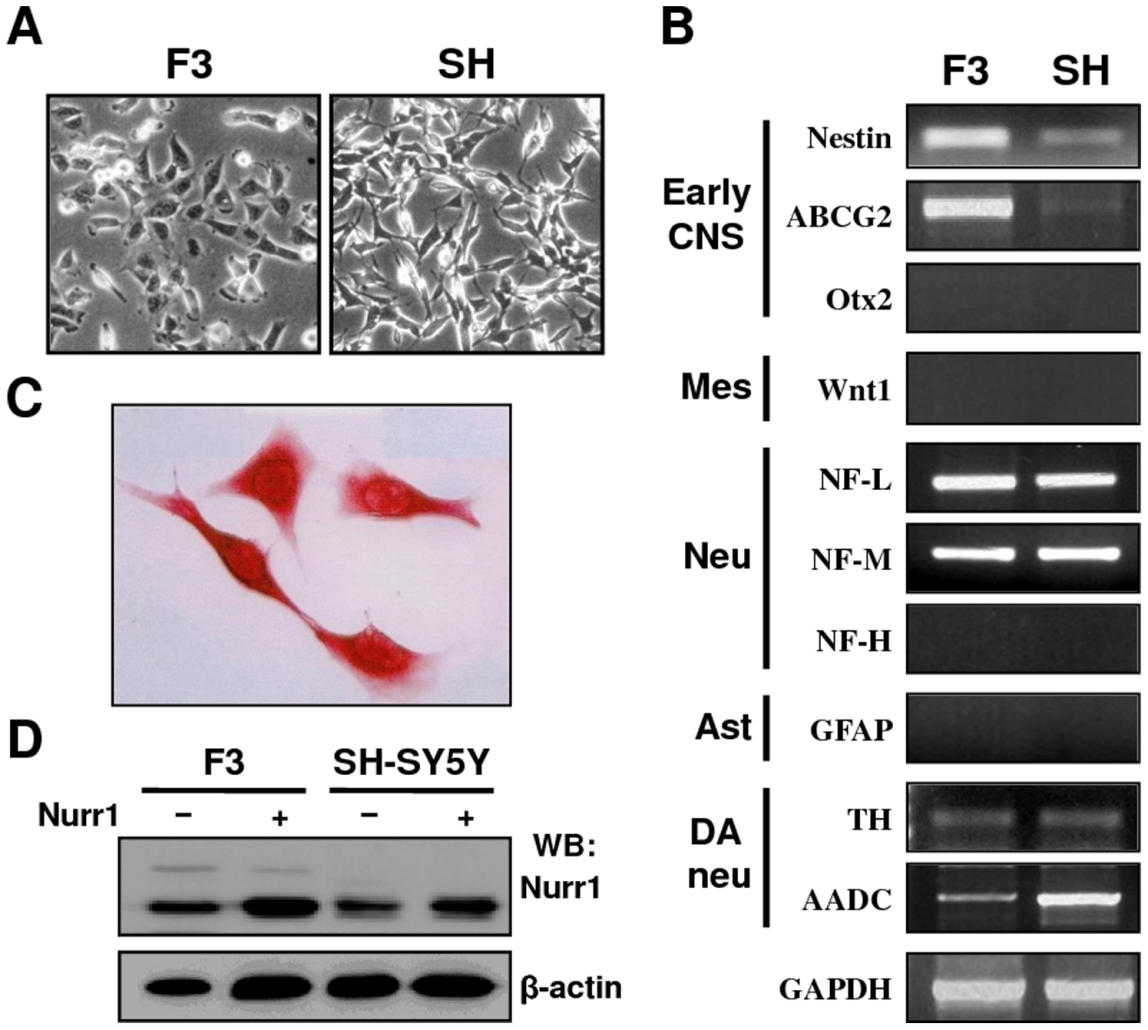
Expression of lineage-specific markers in HB1.F3 and SH-SY5Y cells. (A) Morphology of the hNSC line HB1.F3 and the DA neuron-like SH-SY5Y cells. (B) Semi-quantitative RT-PCR analysis of the early CNS, mesencephalic, neuronal, astrocyte, and dopaminergic markers in HB1.F3 and SH-SY5Y cells. (C) Immunocytochemical analysis of the NSC marker nestin in HB1.F3 cells. (D) Western blot analysis of whole extracts from HB1.F3 and SH-SY5Y cells. Cells were transfected with pLPCX or a Nurr1 expression vector, and cell lysates were subjected to immunoblotting with anti-Nurr1 (upper panel) and β-actin (internal control).

In the present study, we used two cell lines of human origin: HB1.F3 and SH-SY5Y cells (Figure 1 A). HB1.F3 is an immortalized human neural stem cell (hNSC) line derived from human mesencephalon [24], [25]; it has the ability to self-renew and differentiate into cells of neuronal and glial lineages both in vivo and in vitro [26], [27]. The dopaminergic neuron-like SH-SY5Y cells are of human neuroblastoma origin, and can develop a DA neuronal phenotype following stimulation with retinoic acid (RA), phorbol-12-myristate-13-acetate (PMA), or forskolin [28], [29]; these cells are considered a suitable in vitro model for neuronal differentiation [30].

To gain more insight into the molecular mechanism underlying the transcriptional regulation of the hTH gene by Nurr1 and to identify regulatory cofactors that associate with Nurr1 during dopaminergic neurogenesis, we performed promoter mutation and transient transfection assays in hNSCs and neuroblastoma cells. Here, we found that Nurr1 actively represses hTH promoter activity in hNSCs, but it transactivates the hTH promoter in DA cells, suggesting a functional switch for Nurr1 from transcriptional repressor to activator in the development of mdDA neurons. In addition, our findings indicate that SIRT1 is important for DA neuron differentiation, and its spatial regulation may be critical for transcriptional repression of hTH expression in DA precursor cells.

## Materials and Methods

### Cell Culture

The immortalized human NSC lines HB1.F3 and HB1.A4 were established as described previously [24], [25]. The cells were maintained and passaged on uncoated culture dishes in Dulbecco’s modified Eagle’s medium (DMEM, Sigma) containing 10% fetal calf serum (FCS). The human DA neuroblastoma cell line SH-SY5Y was also grown in DMEM supplemented with 10% FCS.

### Plasmid Constructs

To assay hTH promoter activity, a 3301-bp fragment containing the hTH promoter sequence from −3174 to +127 was subcloned directly upstream of the luciferase gene to create phTH-3174-Luc. The selected promoter region contains three consensus elements for Nurr1, NBRE-A, -B, and -C located at positions −2413 to −2406, −1440 to −1433, and −833 to −824, respectively. To localize the NBRE site responsible for transcriptional activity, seven additional constructs were created by serial deletions from phTH-3174 using unique restriction endonuclease sites. All constructs were verified by sequencing. The pSV-β-galactosidase plasmid (Promega) was used as an internal control.

The QuickChange Mutagenesis kit (Stratagene) was used to perform site-directed mutagenesis of the NBRE elements within the hTH promoter. The pGL3-basic-hTH expression vector was used as a template. Oligonucleotides with 34–36 nucleotides and containing the desired point mutations were created as follows, with the mutated residues underlined: mNBRE-A, 5′-GACATTTGCTGCTGAAAAACAGAATCCACATCCGGC-3′; mNBRE-B, 5′-GAAGCAGTTTTAGGAAAAACAGCAGGGGCTATTGTTG-3′; and mNBRE-C, 5′-GAGGAGAAACTGCAAAAACAGCTCCAAGGGGAAGGC-3′. The site-directed mutations were confirmed by sequence analysis. For co-transfection with Nurr1 constructs, we used pLPC-Nurr1, pLPC-Nurr1-a, and pLPC-Nurr1-b plasmids coding cDNA sequences for human full-length Nurr1, Nurr1 splice variant-a (Nu-va), and Nurr1 splicing variant-b (Nu-vb), respectively.

### Transfection and Luciferase Assays

Transfection was performed using Lipofectamine PLUS reagent (Invitrogen) according to the manufacturer’s instructions. A total of 3 µg DNA was used in each transfection. All transfections contained 2 µg luciferase reporter plasmid and 0.5 µg of internal control plasmid pSV-β-galactosidase (Promega). The cells were transfected at 60–80% confluency in 35-mm 6-well plates and harvested 48 h after transfection. Trichostatin A (TSA), butyric acid (BA, sodium butyrate), valproic acid (VPA, 2-propylpentanoic acid), and nicotinamide (NAM) treatments were initiated 24 h after transfection. All transfections were carried out in triplicate. Promoter activity was determined using the Single-LuciferaseTM Reporter Assay System (Promega) following the manufacturer’s recommendations. The luciferase activity was normalized based on the β-galactosidase activity in each well. Statistical analysis was performed using GraphPAD Instat, version 1.13 (Graph Pad Software, San Diego, CA).

### Electrophoretic Mobility Shift Assay (EMSA)

**Figure 2:**
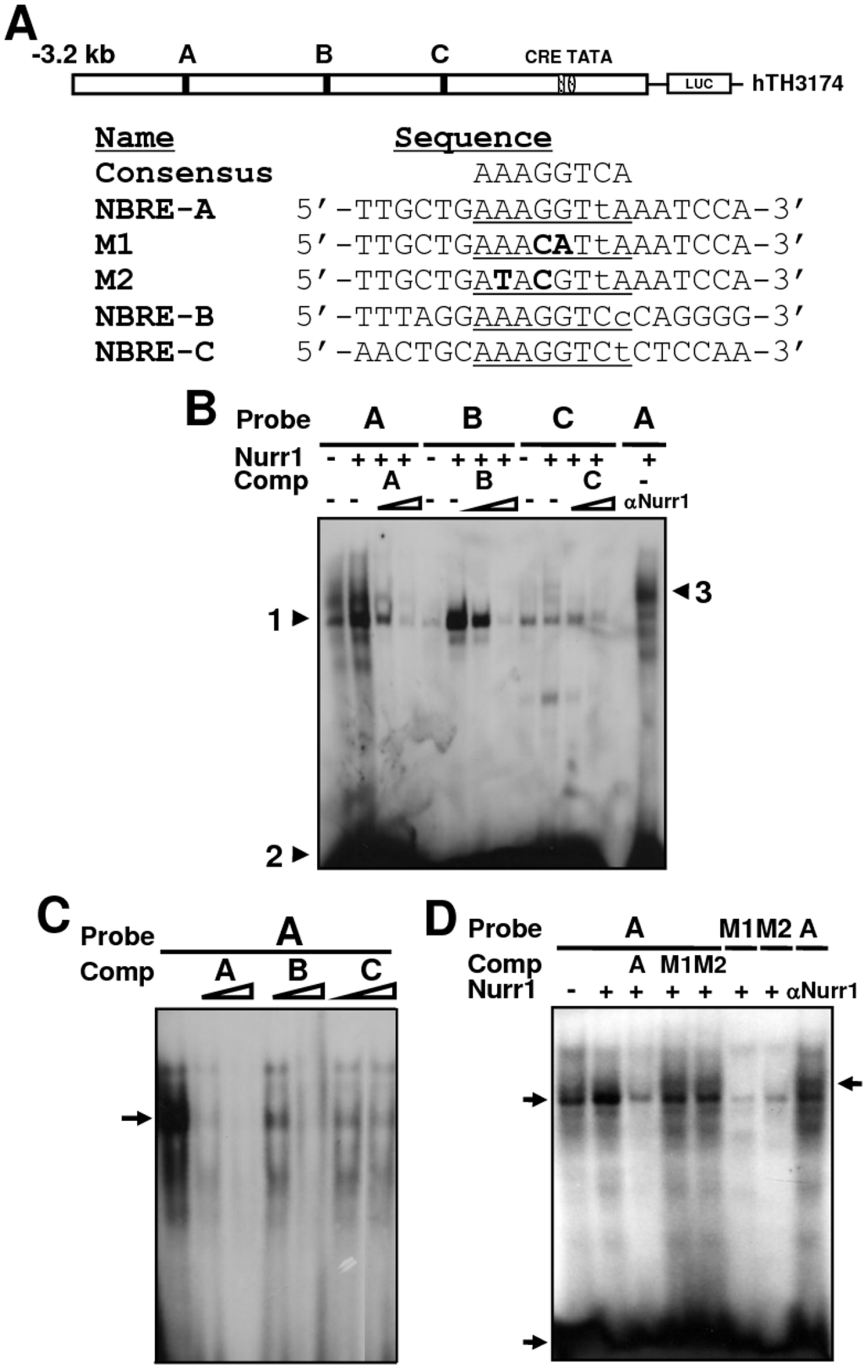
Sequence-specific binding activity of Nurr1 to human TH NBREs. (A) Schematics of the human TH promoter and NBRE oligonucleotides used in this study. The NBRE sites are underlined and mutations are shown in bold. (B) EMSA with ^32^P-labeled oligonucleotides containing human NBREs using nuclear extracts from SH-SY5Y cells. A slow-migrating complex (arrowhead 1) was detected, compared to free-probe migration (arrowhead 2). This pattern was supershifted in the presence of Nurr1 antibodies (arrowhead 3). The DNA-binding activity in the presence of the three TH NBREs was competed out by adding a 40- or 80-fold molar excess of each unlabeled NBRE oligonucleotide. (C) Competition assays between the NBRE-A and NBRE-B or -C sites. Nuclear extract from SH-SY5Y cells transiently transfected with the pLPC-Nurr1 plasmid was incubated with radiolabeled NBRE-A DNA in the presence or absence of 40- or 80-fold molar excess of competitor DNA as indicated above the lanes. (D) The DNA-binding activity in the presence of NBRE-A was competed out by adding a 40- or 80-fold molar excess of unlabeled wild-type oligonucleotide (A) but not by adding mutated NBREs (M1 or M2).

EMSA was performed as described previously [31]. Approximately 10 µg of nuclear extract was used in each reaction. The anti-Nurr1 antibody used in the supershift assays was supplied by Santa Cruz Biotechnology, and the assays were performed according to the manufacturer’s protocol. Sense and antisense oligonucleotides were annealed and end-labeled with [γ-^32^P]dATP (Amersham) and T4 polynucleotide kinase according to standard protocol. All DNA-binding sites were purified on 19% non-denaturing polyacrylamide gels. The oligonucleotide sequences of the radiolabeled probes are provided in Figure 2 A. In standard reactions, proteins and labeled DNA were incubated in a total volume of 20 µl. Reactions were incubated on ice for 15 min prior to loading onto a 5% non-denaturing polyacrylamide gel. The gels were then fixed, dried, and visualized by autoradiography.

### Immunocytochemical Analysis

Cells were fixed with 4% paraformaldehyde for 20 min at room temperature before immunocytochemical staining. Fixed cultures were incubated with rabbit polyclonal anti-SIRT1 antibody (1∶500, Upstate, Lake Placid, NY) at 4°C overnight. After washing, cultures were incubated for 1 hr at room temperature with FITC-conjugated secondary antibody (1∶350, Vector Laboratories, Burlingame, CA) in PBS containing 3% bovine serum albumin. The aclars (SPL Life Sciences, Korea) were then mounted on a glass slide with Vectashield (Vector Laboratories, Burlingame, CA). Fluorescence images were obtained by a confocal microscope (LSM 710, Olympus, Japan).

### Chromatin Immunoprecipitation (ChIP)

The ChIP assay was performed using the EZ-ChIP™ ChIP kit (Upstate) following the manufacturer’s instructions. Briefly, cells were cross-linked by adding 1% formaldehyde and incubating at 37°C for 10 min. They were then washed twice with ice-cold PBS, scraped, and resuspended in SDS lysis buffer. Chromatin was sonicated to an average length of 0.5 kb. Chromatin extracts were diluted 10-fold in dilution buffer, and then pre-cleared with protein G-agarose at 4°C for 1 h with rotation. After pelleting the agarose by brief centrifugation, 2 mg of anti-SIRT1 antibody (Millipore, #07-131) or anti-Nurr1 antibody (TransCruz, sc-991 X) was added to the supernatant fraction and the mixture was incubated overnight at 4°C. We next added 50 µL protein G-agarose, and incubated this mixture at 4°C for 1 h to collect the antibody/antigen–DNA complexes. The chromatin bound to the protein G-agarose beads was eluted with freshly prepared elution buffer (1% SDS and 0.1 M NaHCO_3_). After reversing the cross-linking, DNA was purified using a spin column, and analyzed by PCR for the presence of hTH promoter DNA between −2442 and −23134 bp upstream of the hTH ATG start codon. A 129-bp PCR product was generated using the following primers: forward, 5′-GAAAGCACAACTGGCCCGGCAGG-3′ (−2442); and reverse, 5′-CTGATGACCACCACGCCGGAGGC-3′ (−2313).

## Results

### Nurr1 Directly Binds the NBRE-like Sites in the Human TH Promoter

We analyzed the expressions of neural stem cell markers in HB1.F3 and SH-SY5Y cells (Figure 1 B). HB1.F3 cells were strongly positive for several markers, including the early CNS markers nestin and ATP-binding cassette sub-family G member 2 (ABCG2), but were negative for the mesencephalic marker Wnt1, the astrocyte marker glial fibrillary acidic protein (GFAP), and the terminally differentiated neuronal marker neurofilament heavy polypeptide (NF-H) (Figure 1 B–C). HB1.F3 cells also weakly expressed the DA markers TH and aromatic L-amino acid decarboxylase (AADC) (Figure 1 B). Similar findings were obtained in all experiments, regardless of whether the cells were from early or late passages. The expression profile indicated that the HB1.F3 cells expressed NSC markers. In contrast, SH-SY5Y cells did not express NSC markers at all. Compared to the HB1.F3 cells, the SH-SY5Y cells expressed the DA markers TH and AADC more strongly. Additionally, immunoblotting of the cell extracts with anti-Nurr1 antibody showed two endogenous immunoreactive bands: a major band of about 72 kDa, corresponding to the expected unmodified Nurr1; and a less intense and more slowly migrating band of about 95 kDa. The 95-kDa band was observed only in the hNSC line, not in SH-SY5Y cells (Figure 1 D). A recent report suggests that the 95-kDa slower-migrating band may correspond to the SUMOylated form of Nurr1 [32].

Transcription factor Nurr1 was characterized by binding as a monomer to NBRE sequence motif, heterodimer with retinoid X receptor (RXR) to DR5 or a dimer to NurRE. To determine whether Nurr1 transactivates the hTH promoter by interacting with binding motif(s), we searched the 5′ flanking sequences for the potential Nurr1-binding sequence motifs. No DR5-like or palindromic NurRE motifs were found within the 3.2 kb upstream region, hTH-3174, as we reported previously [16]. However, three NBRE-like motifs with no more than one base deviation from the consensus NBRE were identified in the same promoter region and termed NBRE-A, NBRE-B, and NBRE-C from the distal site at −2315 to −2308 bp, −1452 to −1445 bp, and −837 to −830 bp, respectively.

We used EMSA to establish whether these NBRE sites are able to recruit Nurr1 transcription factor. Oligonucleotides encompassing each NBRE site were used as radiolabeled probes (Figure 2 A). When the NBRE-A oligonucleotide was incubated with the nuclear extracts of DA neuroblastoma SH-SY5Y cells transfected with pLPC-Nurr1 plasmids, a specific retarded complex was observed (Figure 2 B, lane 2). However, complex formation was abrogated in the presence of 40-fold or 80-fold molar excess of cold NBRE-A oligonucleotide (Figure 2 B, lanes 3 and 4). In addition, many other DNA-protein complexes were clearly detected (Figure 2 B lane 1–4). In contrast, both the NBRE-B and NBRE-C probes generated a single major band (Figure 2 B lanes 5–12), though the binding of NBRE-C was very weak (Figure 2 B, lanes 9–12). In addition, we confirmed by supershifting with Nurr1 antiserum that the band observed with the NBRE probes did contain Nurr1 (Figure 2 B, last lane). In addition, the patterns of protein complex formation in hNSCs were similar to SH-SY5Y cells, though Nurr1 binding was relatively weak (Figure S1 in File S1). Taken together, the data clearly demonstrate that NBRE-A and NBRE-B in the hTH promoter can be bound specifically by Nurr1.

To determine whether the NBRE sites had cross-binding affinity, we performed competition assays using increasing amounts of unlabeled NBRE-A, NBRE-B, and NBRE-C oligonucleotides. Though 100-fold excess of unlabeled NBRE-A almost completely inhibited formation of its own DNA-protein complexes (Figure 2 C, lanes 2 and 3), more than 100-fold excess of NBRE-B or NBRE-C was required for the same level of interference (Figure 2 C, lanes 4 and 7). In addition, Nurr1 binding to the NBRE-B or NBRE-C site was successfully inhibited by the NBRE-A oligonucleotide (Figure S2 in File S1). This result indicates that NBRE-A binds to Nurr1 with high affinity compared to NBRE-B or NBRE-C. To characterize the DNA-protein interaction at the NBRE-A site in greater detail, we performed EMSA and competition assays with mutated probes. Because Nurr1 does not bind to the mutated NBRE in which the second, fourth, and fifth nucleotides are replaced [33], we introduced the corresponding mutation into the NBRE-A sequence. The mutated probes mNBRE-A1 and mNBRE-A2 could not form the retarded complex with Nurr1 (Figure 2 D, lanes 6 and 7). The addition of unlabeled, mutated probes mNBRE-A1 or mNBRE-A2 did not inhibit the binding of Nurr1 to NBRE-A (Figure 2 D, lanes 4 and 5), whereas addition of unlabeled probe NBRE-A did inhibit binding (Figure 2 D, lane 3). Similar binding patterns were shown for the NBRE-B and NBRE-C sites (Figure S3 in File S1). These results strongly suggest that Nurr1 binds directly to the hTH-NBRE-A sequence.

### Nurr1 Functions as a Dual Function Transcription Factor in hTH Expression

**Figure 3:**
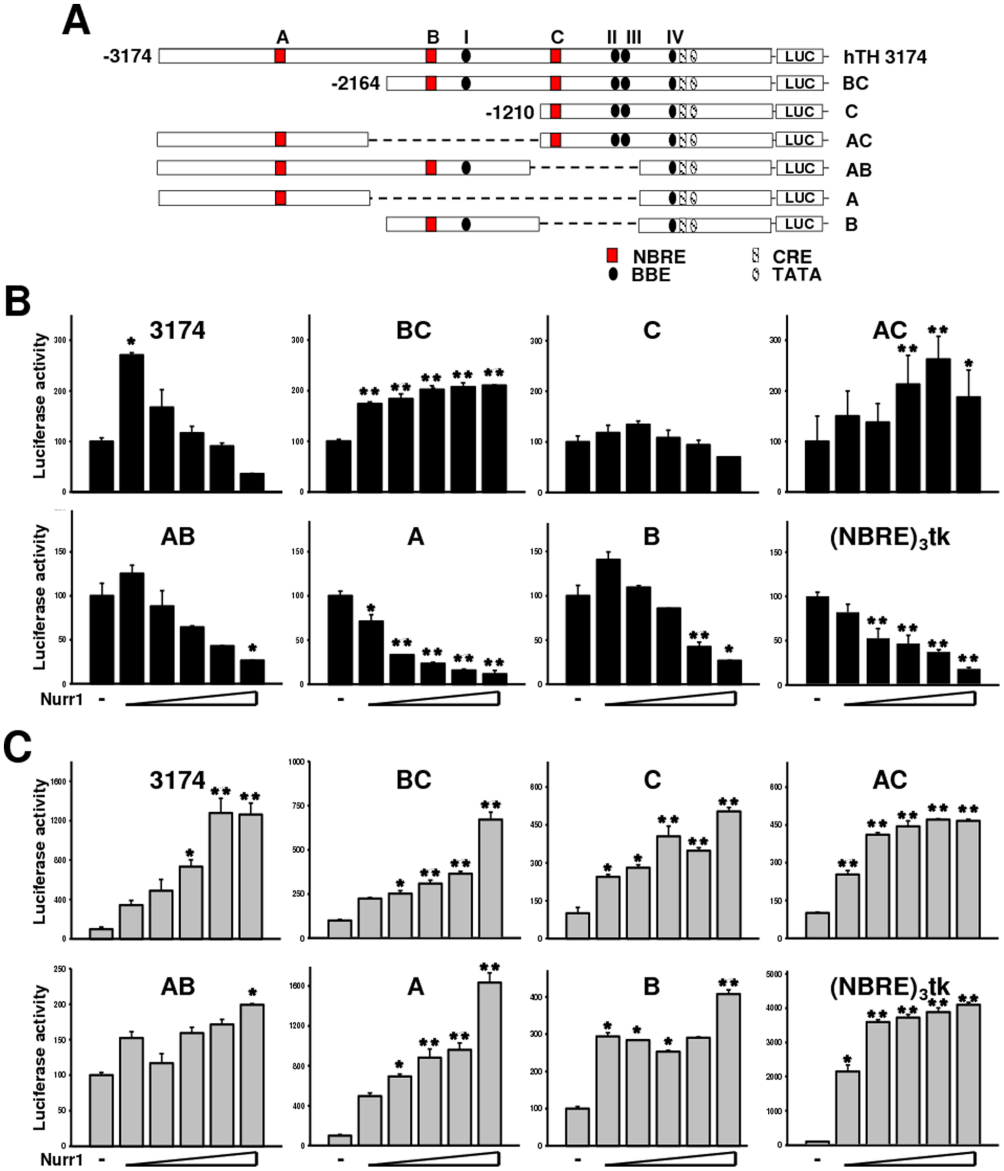
Transactivation of the human TH gene promoter by Nurr1. (A) Schematic of the human TH promoter deletion constructs. The locations of NBRE sites A–C, the CRE site, and TATA box are shown. The longest promoter fragment starts at –3174 bp, and all of the constructs have common 3′-ends at +145 bp with respect to the transcriptional initiation site. (B and C) Responsiveness of the TH promoters to Nurr1 in F3 (B) and SH-SY5Y cells (C). Each deletion construct of phTH3174 to phTH-B and pSV-β-gal was transfected into F3 and SH-SY5Y cells in the presence or absence of pLPCNurr1. To compare Nurr1 transactivation activities directly, the luciferase activity of each reporter construct in the presence of empty vector (pLPCX) was set to 100%. The figure represents the mean ±S.D. (bar) value of triplicate samples in a representative experiment. The experiments were repeated three times with similar results.

To grossly characterize the Nurr1 responsiveness of the hTH promoter, a series of promoter deletion mutants were generated from the hTH-3174 construct, designated TH-BC, TH-C, TH-AC, TH-AB, TH-A, and TH-B (Figure 3 A). Each construct or a synthetic reporter construct with trimerized NBRE sites upstream of a thymidine kinase promoter, p(NBRE)_3_-tk [33] with or without pLPC-Nurr1, was transiently transfected into hNSC line HB1.F3 and SH-SY5Y cells, and the reporter assay performed. As shown in Figure 3 C, Nurr1 stimulated the hTH-3174 promoter activity in DA-like cells in a dose-dependent manner. Interestingly, the same amount of Nurr1 resulted in the biphasic pattern of promoter activity in F3 cells: transactivation at a low dose but transrepression at a high dose (Figure 3 B). The Nurr1 responsiveness of the deletion constructs also showed a marked increase (2-fold to 16-fold) in SH-SY5Y cells, similar to that of hTH-3174. However, in HB1.F3 cells, TH-AB, TH-A, and TH-B showed strong transcriptional repression, but not the other deletion constructs. We also extended the luciferase-based reporter assay to a synthetic Nurr1-responsive promoter, (NBRE)_3_-tk. The Nurr1 expression vector impaired transcription of the synthetic promoter more dramatically than the hTH-3174 reporter in F3 cells (Figure 3 B). In contrast, (NBRE)_3_-tk was stimulated robustly by Nurr1 in SH-SY5Y cells (Figure 3 C). The activity of (NBRE)_3_-tk showed a similar pattern as the TH-A reporter in both cells. Among all of the constructs we tested, TH-A exhibited the highest responsiveness to Nurr1 in both SH-SY5Y and F3 cells, strongly suggesting that NBRE-A is involved in transcriptional regulation of the hTH promoter.

### NBRE-A is a Critical cis-acting Element for Transcription of the hTH Promoter by Nurr1

**Figure 4:**
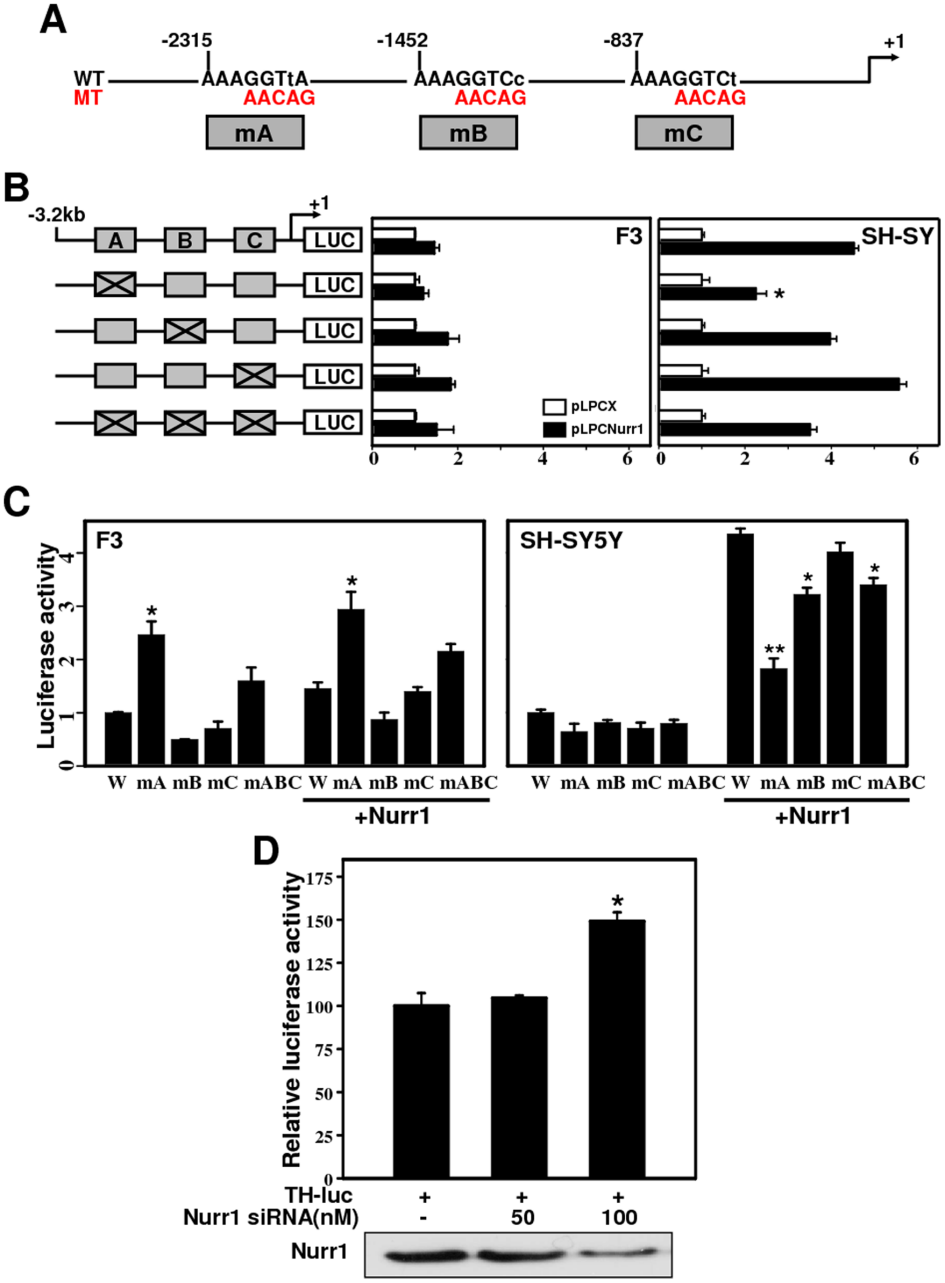
The NBRE-A site is critical for Nurr1-mediated transcriptional regulation of the TH promoter. **(**A) Schematic of the TH promoter encompassing three putative NBREs. The sequences of the putative NBRE motifs are shown, with lowercase letters representing the nucleotides deviating from the consensus NBRE motif. (B) Putative NBRE motifs are shown as gray boxes; gray boxes marked with X represent a mutation of the putative NBRE motif to AAAAACAG. The indicated constructs were transiently co-transfected into cells with either Nurr1 expression plasmid (pLPCNurr1) or empty plasmid. The activation is expressed relative to the luciferase activity obtained after co-transfection of pLPCX, which was assigned a value of 1 for each construct. (C) The same data from (B), plotted relative to the wild-type TH promoter construct, which was set arbitrarily to 1. *P<0.05. (D) Effects of siRNA targeting the Nurr1 genein HB1.F3 cells. Cells were transfected with the indicated amount of Nurr1 siRNA. Luciferase activit*y was* analyzed 48 h after siRNA transfection. *P<0.001.Bottom panel, knockdown of Nurr1 was assessed by Western blot.

Based on our deletion analysis indicating that the upstream region encompassing −3.2 to −2.2 kb retains most of the responsiveness to Nurr1 transactivation (Figure 3), we hypothesized that NBRE-A may be a critical cis-regulatory element for transcription of the hTH gene by Nurr1. We mutagenized three NBRE-like sites in the context of the 3.2-kb upstream sequences and determined the promoter activity in F3 and SH-SY5Y cells (Figure 4 A–C). In SH-SY5Y cells, co-transfection of Nurr1 stimulated the wild-type promoter 4.5-fold (Figure 4 B, right panel). When only the NBRE-A site was mutated, activation of the promoter by Nurr1 was reduced dramatically to 35.7% of the wild-type (Figure 4 B). However, mutation of the NBRE-B or NBRE-C site had little effect on Nurr1 transactivation of the promoter. In contrast to SH-SY5Y cells, HB1.F3 cells exhibited relatively little effect on the transactivation of hTH promoter activity in any of the mutant constructs (Figure 4 B, middle panel). These data clearly demonstrate that the NBRE-A site is essential for full transcriptional activation of the hTH promoter in SH-SY5Y cells, but not F3 cells, and that the other two NBRE sites do not make a significant contribution.

Next, we compared the basal promoter activity of NBRE mutant constructs (Figure 4 C). Though the basal activity of the wild-type hTH promoter was increased two- to three-fold by mutating the NBRE-A site in hNSC line HB1.F3, no change was found in SH-SY5Y cells. However, in the hTH promoter harboring NBRE-B or NBRE-C site mutation, neglectable changes in transcriptional activity occurred in both cell lines. The transactivational or repressional activity of the triple mutant appeared to be the sum of each mutant’s activity, suggesting complementary functions of the other two NBRE sites. Mutant promoter studies established the importance of the NBRE-A site for Nurr1-mediated hTH promoter activation and repression in SH-SY5Y and F3 cells, respectively.

These results raise the possibility that endogenous Nurr1 or other protein(s) directly binds to NBRE-A and mediate repression in HB1.F3 cells, but not SH-SY5Y cells. As HB1.F3 cells express high endogenous levels of Nurr1, we knocked-down Nurr1 in HB1.F3 cells using siRNA strategy to evaluate the impact of Nurr1 on the repression of hTH promoter activity (Figure 4 D). Immunoblot analyses confirmed significant down-regulation of Nurr1 expression in HB1.F3 cells transfected with Nurr1 siRNA target sequences (Figure 4 D, bottom panel). Next, we performed luciferase assays using the hTH-3174 promoter in the presence of increasing concentrations of siRNA to evaluate the role of Nurr1 in hTH expression. Knockdown of Nurr1 expression increased hTH promoter activity compared to control HB1.F3 cells, and the maximum activity was observed at a siRNA concentration of 100 nM (Figure 4 D, upper panel). These results indicate that Nurr1 may function as a dual function transcription factor that recruits co-repressors in hNSCs or co-activators in DA-like cells and forms a complex at the NBRE-A site of the hTH promoter.

### The N-terminus of Nurr1 Contains the Major Repression Function

**Figure 5:**
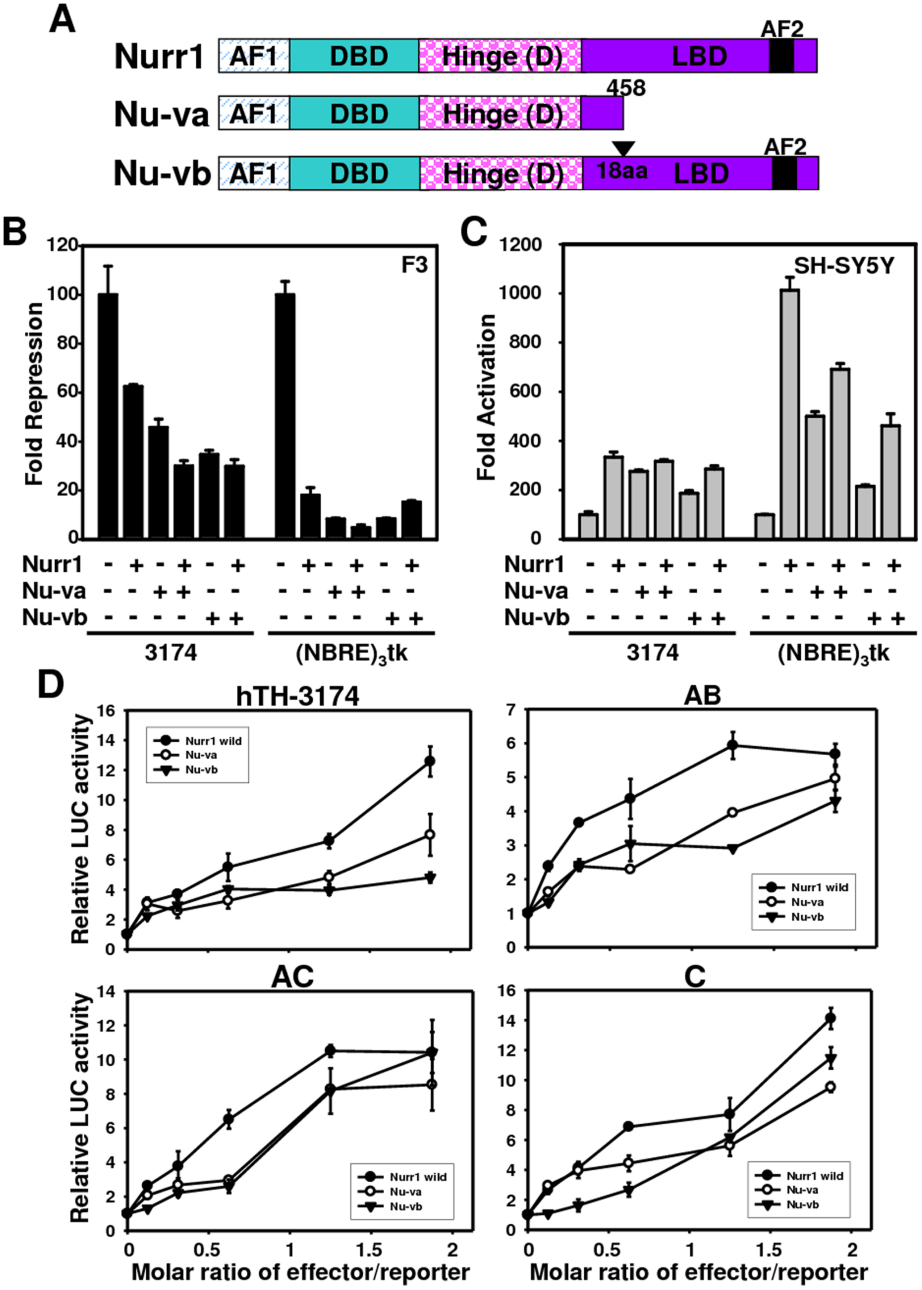
The dual functions of Nurr1 in NBRE-A site-mediated TH gene expression. (A) Schematic of the structure of Nurr1-wild and its splice variants. AF1 or AF2, transactivating domain 1 or 2; DBD, DNA binding domain; LBD, ligand binding domain. (B and C) Transcriptional regulation of the TH gene by Nurr1 splice variants. F3 (B) and SH-SY5Y cells (C) were transfected with either hTH-3174 or NBRE-3x-TK reporter construct in the absence or presence of Nurr1 splice variants. Experiments were repeated at least three times. Results are shown as mean ± S.D. (D) Responsiveness of hTH luciferase reporter genes to variant Nurr1 proteins. The reporter plasmid hTH-Luc was co-transfected with the effector plasmid pLPCX-Nurr1, pLPCX-Nurr1-variant A, or pLPCX-Nurr1-variant B into SH-SY5Y cells. The amount of effector plasmid transfected in each experiment is shown at the bottom as the molar ratio of effector plasmid to reporter plasmid. In each experiment, stimulation of reporter gene expression by co-transfected effector plasmid is presented as the fold induction using the average value from three independent samples.

Ichinose et al. [34] reported the presence of two types of Nurr1 splice variants in fetal human brain as in the Figure 5 A. Nu-va is a 455-amino-acid protein with a truncated carboxy terminal region, whereas the wild-type Nurr1 protein is 598 amino acids. Nu-vb is a protein consisting of 580 amino acids with a deletion of 18 amino acids within the carboxy terminal ligand-binding domain [34], [35]. Alternative splicing could control Nurr1 function in a more complicated fashion. Therefore, we determined whether the human Nurr1 variants have biological activity. Various luciferase reporter expression constructs were used in transient co-transfection experiments with Nurr1 protein expression constructs to determine the transactivation and/or transrepression activities of the Nurr1 variants compared to full length Nurr1. When full-length Nurr1 was co-transfected into HB1.F3 cells with p(NBRE)_3_-tk, the activity was reduced by approximately one-fifth (Figure 5 B). Both splice variants (Nu-va and Nu-vb) reduced the reporter activity more than full-length Nurr1 (approximately 10% of the hTH promoter activity). The promoter activity of the hTH-3174 reporter was repressed in a similar manner, though the reduction was less than that of p(NBRE)_3_-tk (Nurr1, 63%; Nurr1a, 46.3%; Nurr1b, 35.2% of the hTH promoter activity). This result indicates that the N-terminus of Nurr1 contains the major repression function independent of a Nurr1 ligand.

In contrast, all types of Nurr1 proteins functioned as transcriptional activators in SH-SY5Y cells, though the rate was different between constructs (Figure 5 C-D). Co-transfection of the full-length Nurr1 expression plasmid resulted in 3.5-fold activation of the hTH reporter and 11-fold activation of p(NBRE)_3_-tk, demonstrating constitutive transcriptional activity of full-length Nurr1 in SH-SY5Y cells (Figure 5 C). The SH-SY5Y cells transfected with Nu-va and Nu-vb exhibited a significantly lower level of luciferase activity (59.7% and 44.8% with the phTH-3174-Luc construct and 52.9% and 25.8% with p(NBRE)_3_-tk) compared to cells transfected with full-length Nurr1 (Figure 5 C). To further investigate whether Nurr1 protein variants could activate the hTH promoter in SH-SY5Y cells, increasing amounts of a Nurr1 splicing variant expression plasmids were co-transfected with the hTH promoter plasmids (Figure 5 D). In transactivation of the hTH-3174 promoter, transfection with the cDNAs of both Nurr1 protein variants caused less transactivation than the full-length Nurr1, with Nu-vb exhibiting the lowest activity (Figure 5 D). However, transfection with Nu-va or Nu-vb cDNAs resulted in similar activity in the transactivation of the AB, AC, and C promoters. Activation was approximately halved for all promoter constructs in the presence of Nu-va and Nu-vb cDNAs compared to full-length Nurr1 (Figure 5 D). All of these observations suggest that the Nurr1 protein variants with LBD deletions are able to mediate a specific transcriptional response via NBRE, but that they are unexpectedly more active in transcriptional repression than the full-length Nurr1 protein. However, our results for transcriptional activation are consistent with reports in other cell types [35], [36], [37] that the absence of the Nurr1 C-terminal decreases the activation of p(NBRE)_3_-tk or the TH reporter in SWI353 cells. Because of their different transcriptional properties compared to full-length Nurr1 in regards to both repression and activation, proteins coded by these transcripts could potentially function as partial competitors in the regulation of the expression of Nurr1 target genes.

### Nurr1-mediated Repression is Sensitive to SIRT1 Inhibitor Nicotinamide

**Figure 6:**
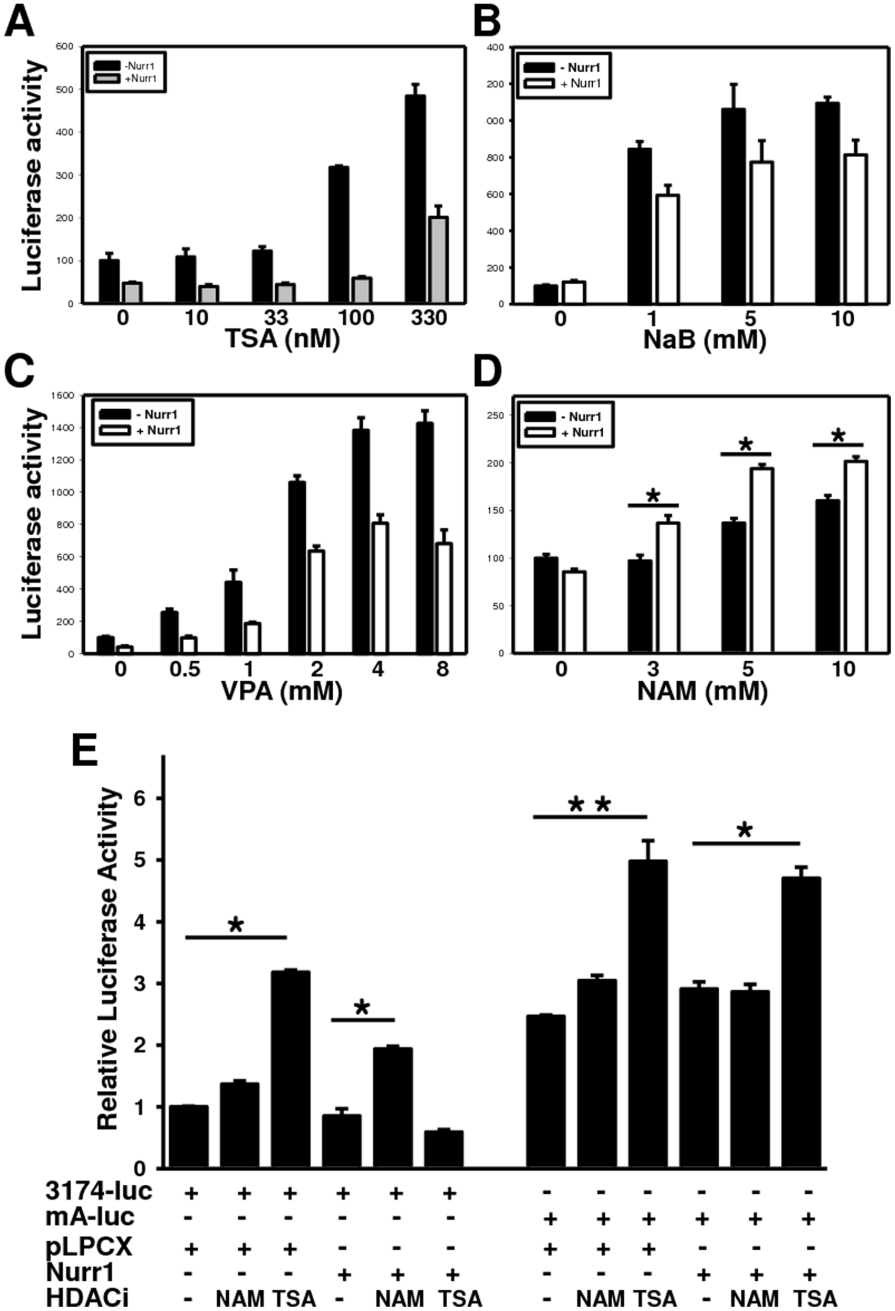
A class III HDAC is required for Nurr1-mediated suppression of TH gene transcription. (A–D) The TH reporter construct was transiently co-transfected into F3 cells with the pLPCX (closed bar) or pLPC-Nurr1 (gray bar) plasmids, and luciferase activity was determined after 24 h of treatment with TSA (A), NaB (B), VPA (C), or NAM (D). The mean activity of cells transfected with only reporter construct was set to 100%. (E) Identification of NBRE-A as a direct target of both Nurr1 and class III HDAC. Luciferase assays were performed using F3 cells transfected with the hTH-3174 or mA luciferase reporter, with or without the indicated expression vectors (200 ng; full-length Nurr1 or parent vector), and in the presence or absence of histone deacetylase inhibitors, TSA (100 ng/ml), or nicotinamide (NAM, 10 mM). Data were normalized to values for parental cells expressing only the wild-type hTH-luciferase construct (first bar). *P<0.05.

The inhibitory effects of Nurr1 on hTH promoter activity suggest that Nurr1 functions as a transcriptional repressor of hTH promoter activity in hNSCs. Because transcriptional repressors often recruit histone deacetylases (HDACs) to transcriptional complexes, we evaluated whether the inhibition of hTH promoter activity by Nurr1 requires HDAC activity (Figure 6). After transfecting HB1.F3 cells with hTH-3174 and the Nurr1 expression plasmid, the cells were subsequently treated with and without TSA, NaB, VPA (inhibitors of class I and II HDACs) [38], [39], or nicotinamide (NAM, an inhibitor of the SIRT family of HDACs) [40] (Figure 6 D). For basal expression of the hTH promoter, all class I and II HDAC inhibitors, including TSA, NaB, and VPA, superinduced this expression in a dose-dependent manner (Figure 6 A–C). However, these inhibitors could not rescue the transcriptional repression by Nurr1. NAM had little effect on the basal hTH expression (Figure 6 D), but it relieved the Nurr1-mediated repression of hTH promoter activity. These findings show that Nurr1 represses hTH promoter activity in a manner dependent on the SIRT activity in hNSCs.

As shown in Figure 6 D, NAM treatment led to relieve the repression of the activity of hTH promoter containing an intact NBRE-A. We investigated whether the effect of NAM is directly mediated through NBRE-A within the promoter (Figure 6 E). Cells were also transiently transfected with hTH-3174 or the mA-luc reporter construct in the presence of NAM or TSA. We did not observe any significant differences in luciferase activity between NAM-treated and non-treated cells when NBRE-A was mutated, but the repression of the hTH-3174-luc promoter activity was reduced approximately 2-fold (Figure 6 E). In contrast to NAM, the effect of TSA was not significantly different between the two promoters. These results suggest that the SIRT1-mediated repression relieved by NAM treatment is dependent on the presence of an intact NBRE-A.

### Nurr1 Enhances SIRT Recruitment on the NBRE-A Region of the hTH Promoter

**Figure 7:**
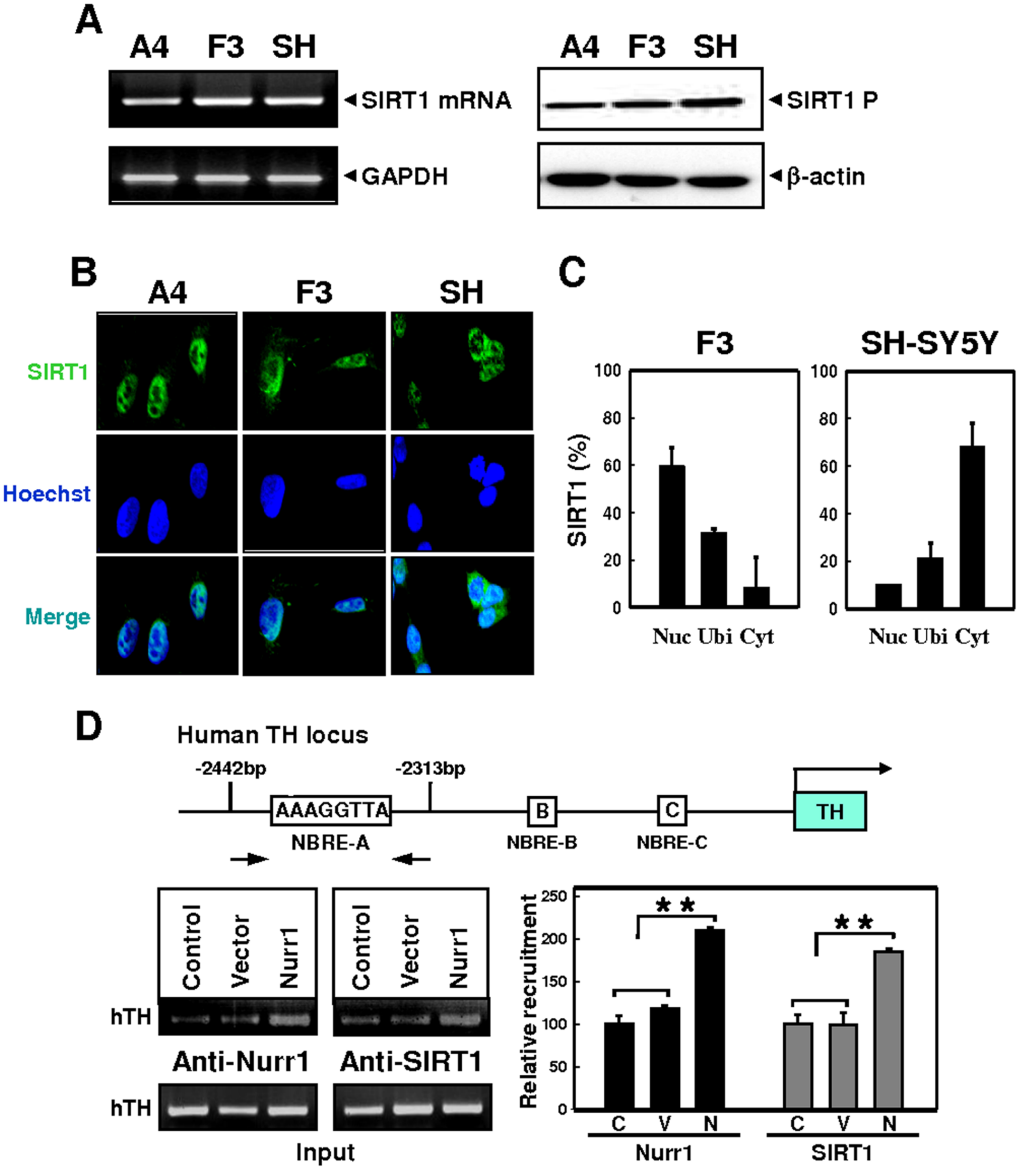
SIRT1 associates with the hTH promoter in an hNSC-specific manner. (A) Expression levels of SIRT1. SIRT1 mRNAs (left panel) and proteins (right panel) were detected by RT-PCR and immunoblotting with anti-SIRT antibody in the hNSC lines HB1.F3 and HB1.A4, and in SH-SY5Y cells. (B) Immunofluorescent images in HB1.F3, HB1.A4 (left), and SH-SY5Y cells (right), using anti-SIRT1 antibody (green). Nuclei were stained with DAPI (blue). (C) Quantification of SIRT1 localization in HB1.F3 and SH-SY5Y cells. Localization was scored as nuclear, cytoplasmic, or ubiquitous (mean±se; n = 86 for HB1.F3 and n = 117 for SH-SY5Y cells). (D) Binding of SIRT1 to the hTH promoter was evaluated by ChIP assay in HB1.F3 cells transfected with no DNA (Control), pLPCX (Vector), or pLPC-Nurr1 (Nurr1). DNA fragments covering NBRE-A on the hTH promoter are indicated in the upper panel. Data are from one representative experiment of three. **P<0.001, Student’s *t*-test.

Nucleo-cytoplasmic SIRT1 trafficking is involved in the regulation of cellular differentiation and gene expression [41], [42]. To investigate the mechanism underlying the differential regulation of hTH expression in HB1.F3 and SH-SY5Y cells, we first examined the subcellular SIRT1 distribution in both cell lines (Figure 7). SIRT1 expression was similar in both cell types (Figure 7 A); however, immunostaining of endogenous SIRT1 revealed significantly different subcellular localizations in the two cell lines (Figure 7 B–C). SIRT1 was principally localized in the nucleus in >90% of HB1.F3 cells, while ∼70% of SH-SY5Y cells only expressed SIRT1 in the cytoplasm (n = 100 for each group; Figure 7 B–C).

Since Nurr1-induced hTH repression was sensitive to SIRT1 inhibition (Figure 6 D–E) and SIRT1 was preferentially located in the nucleus in HB1.F3 cells (Figure 7 B–C), we speculated that SIRT1 recruitment to the hTH promoter might lead to hNSC-specific repression of hTH promoter activity by Nurr1. ChIP assays showed that control and vector-transfected HB1.F3 cells exhibited low levels of SIRT1 on the NBRE-A region of the hTH promoter (Figure 7 D, lower panel). The occupancy of SIRT was significantly increased by transfection of the Nurr1 expression plasmid (Figure 7 D, lower panel), coinciding with the increased association between Nurr1 and SIRT1 in hNSCs. In contrast, no significant SIRT1 recruitment was observed in SH-SY5Y cells (Figure S4 in File S1). Overall, our data suggest that SIRT1 nuclear accumulation is coupled to Nurr1-mediated hTH transcription repression, and that SIRT1 nucleo-cytoplasmic shuttling may play a role in hTH gene expression during DA neurogenesis.

## Discussion

Nurr1 is an orphan nuclear receptor best known for its essential roles in the development and maintenance of mdDA neurons, which regulate motor control and degenerate during Parkinson’s disease. During DA neurogenesis, Nurr1 directly targets TH [9], [10]. Here we investigated the transcriptional mechanisms by which Nurr1 regulates human TH expression. We previously found that the hTH-3174 promoter contains no DR5-like or palindromic sequence motifs, but it has three NBRE-like motifs (NBRE-A, -B, and -C) [16]. Our present promoter studies revealed that the distal NBRE-A site was essential for transactivation of the hTH promoter by Nurr1 in SH-SY5Y cells. However, Nurr1 also actively silenced hTH promoter activity in hNSC lines. Both Nurr1-mediated transcriptional activation and repression were mediated through NBRE-A, and the other two NBRE sites did not significantly contribute to any promoter activity. From these data, we conclude that Nurr1 acts as a dual-function transcription factor for hTH: a transrepressor in hNSCs and a transactivator in DA neuronal cells. Additionally, EMSA showed that the NBRE-A site was the most accessible of the three sites, suggesting differential regulation of Nurr1 recruitment. This difference may be controlled by the context of the hTH promoter. Our previous study identified the two regions (CR-1 and CR-2) that are conserved between the human and rodent TH promoter regions [16]. Notably, the NBRE-A site is located within the CR-1 sequence, supporting the idea that NBRE-A plays important roles and is conserved in mammalian TH transcription. Consistent with this finding, Romano et al. [23] reported that, although a low degree of sequence homology exists, the hTH promoter could drive tissue-specific expression in the midbrain of transgenic mice.

Several extracellular signals modulate the transcriptional function of nuclear receptors through the exchange of co-regulators and co-integrators [22], [43], [44], [45]. Similar to other reports, we demonstrated that Nurr1 transactivated hTH expression in DA cells. However, we unexpectedly found that Nurr1 repressed hTH gene expression in hNSCs. Nurr1 may exist in hNSCs in co-repressor complexes that are tonically remodeled to co-activator complexes during DA neurogenesis in response to differentiation cues. It was recently proposed that the C-terminal domain of Nurr1 mediates cell type-specific transactivation [45] or transrepression [46], [47], [48] dependent on tissue-specific cofactors. For example, β-catenin [45] and Pitx3 [46] interact with the C-terminus of Nurr1 and disrupt its association with the transcriptional co-repressors Lef-1 and SMRT, leading to co-activator recruitment and induction of Nurr1-responsive genes. However, the results of our studies with splice variants showed that the C-terminus of human Nurr1 may not be required for repression of hTH gene expression, as all Nurr1 splice variants exhibited stronger transrepressional activity compared to the full-length Nurr1 protein. Thus, Lef-1 and SMRT were excluded as candidates for interacting with Nurr1. Interestingly, several known transcription repressors–including CRIF1, which inhibits Nur77–reportedly bind to the N-terminus of the NR4R family of transcription factors and inhibit their transcriptional activity [48]. It is plausible that co-repressors similar to CRIF1 may interact with and regulate Nurr1 activity in regards to hTH gene expression. Previous results show that promoter activity induction is dependent on the interactions of Nurr1 with NBRE sites [20], [49], yet repression of MMP-1 [44] or aromatase [50] transcription by Nurr1 is not mediated through an NBRE sequence. In contrast, we found that both induction and repression of transcription were dependent on interactions with NBRE sites.

Histone deacetylation is firmly implicated as being involved in transcriptional silencing, possibly by inducing chromatin condensation [47], [51], [52]. Our HDAC inhibitor studies showed that repression of hTH promoter activity in hNSCs occurred with varying sensitivity to HDAC subclass-specific inhibitors. TSA, NaB, and VPA (class I and II HDAC inhibitors) affected the basal promoter activity of hTH expression, without having obvious effects on the repression of hTH expression by Nurr1. This finding led us to speculate that basal expression and Nurr1-mediated repression were differentially regulated in hNSCs. Kim et al. [46] reported that in TH gene activation in rats, the Sp1 and CRE sites in the proximal promoter are the target elements for the HDAC inhibitors TSA and NaB [53]. Interestingly, the hTH promoter contains Sp1 and CRE sites in the proximal conserved region, CR-2 [16], suggesting that these are common class I and II HDAC-regulated elements in both human and rodent TH promoters. Our data showed that TSA, NaB, and VPA activated hTH promoter activity with low Nurr1 expression, supporting the premise that class I and II HDACs may repress basal transcriptional activity through either the Sp1-binding site or CRE sites in the hTH promoter.

Contrary to the findings with class I and II inhibitors, transcriptional repression of Nurr1 was sensitive to NAM, but not TSA, NaB, or VPA in hNSCs. The promoter with a mutated NBRA-A site no longer showed NAM-sensitivity, implying that a class III HDAC inhibitor (i.e., SIRT1) [54] was involved in the NBRE-A-mediated repression of hTH by Nurr1. Nucleo-cytoplasmic trafficking of SIRT1 is involved in controlling cellular differentiation and gene expression [41], [42]. We revealed that SIRT1 was principally localized in the nucleus of hNSCs in which hTH expression was repressed by Nurr1. In contrast, cytoplasmic localization of SIRT1 was primarily observed in DA neuron-like cells. ChIP assays showed prominent binding of SIRT1 on the NBRE-A element of the hTH promoter in an hNSC-specific manner. The nuclear exclusion of SIRT1 and its cytoplasmic localization may physiologically act to modulate the negative regulation of transcription factor Nurr1 by SIRT1 in DA-like cells. This idea is supported by our finding of differential SIRT1 localization in NSC and DA neuron-like cells.

Our HDAC inhibitor study also indicated that two HDAC-regulated elements were present within the upstream 3174 bp of the hTH promoter: CR-1, which contains the NBRE-A element regulated by class III HDACs; and CR-2, which contains Sp1 and CRE sites regulated by class I and II HDACs. These two CRs showed interesting crosstalk in the regulation of hTH transcription depending on the Nurr1 expression level. With low Nurr1 expression, CR-2 regulated TH expression independently of CR-1. However, with Nurr1 over-expression, CR-1 regulated TH expression in a higher hierarchical order. To the best of our knowledge, this study is the first to find that hTH promoter activity is repressed in a manner regulated by Nurr1 and the NAM-sensitive HDAC SIRT1.

We also identified an interesting difference in the mechanisms of TH gene regulation between the human and rodent models. Nurr1 actively repressed hTH promoter activity in hNSCs, but transactivated this activity in DA cells. In contrast, other reports show that Nurr1 actively induces rodent TH expression in both neural precursors and differentiated cells [5], [19], [20]. This discrepancy may be because Nurr1 recruits some yet unidentified cofactor(s), resulting in the high affinity of Nurr1 for NBRE-A and repressing the hTH promoter activity in an hNSC-specific manner. Another possible explanation is that the transcriptional repression may be relieved in a rodent cell culture system in contrast to the effect in an in vivo system. Consistent with this notion, Jacobs et al. [22] reported that, in a mouse model, Nurr1-mediated transcription is repressed in a HDAC-dependent manner in the absence of Pitx3, and that Pitx3 recruitment to the Nurr1 transcriptional complex leads to activation of Nurr1 target genes via induction of SMRT/HDAC release. Because the TH gene is expressed in the final stage of DA neurogenesis, its expression at premature stages might result in abnormal differentiation. The human system may include more elaborate mechanisms to prevent abnormal gene expression and finely tune the transcriptional responses when other neuronal differentiation, such as neurite outgrowth, has not yet matured. Our results suggest that Nurr1 may be a key regulator of DA neurogenesis, playing a role in regulating the timing of the transcriptional activation of the hTH gene. This regulation may also explain why Nurr1 has different functions in hNSCs and DA neuron-like cells.

**Figure 8:**
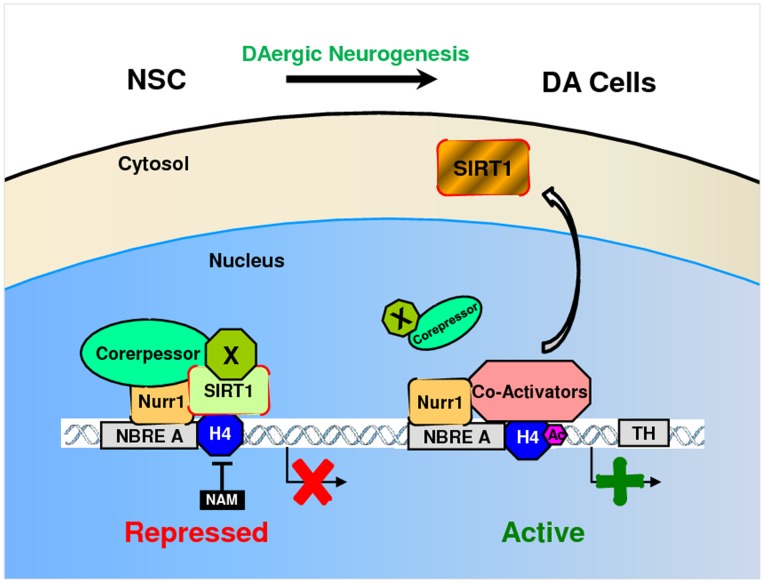
Model of the regulation of the TH gene by the SIRT1 co-repressor complex in the NBRE-A element. In neural precursor cells (top), the TH gene is maintained in a repressed state by the action of a co-repressor complex containing SIRT1 at the NBRE-A element and class I and II HDACs at the CRE and TATA elements, respectively. As hNSCs finish differentiation (bottom), DA-inductive signaling (e.g., Notch or Wnt) switches Nurr1-associated proteins from co-repressors to co-activators at the NBRE-A site. In addition, the class I and II HDACs may be down-regulated or modified to inactive forms, and RNA polymerases replaces HDACs in CRE sites. Both interactions cooperatively induce TH transcription.

From the present results, we propose a working model (Figure 8) in which hTH gene expression is maintained in a repressed state by the action of a co-repressor complex containing Nurr1 and SIRT1 recruited via unknown adaptor factor(s) to the NBRE-A site in the absence of a differentiation cue. The enzymatic activity of SIRT1 removes acetyl residues from the histones in chromatin, inducing chromatin condensation, which represses transcription. In this model, DA differentiation cues stimulate both the transactivation function of Nurr1 and the translocation of SIRT1 to the cytoplasm, leading to a switch in equilibrium toward the co-activator complex and activation of hTH expression.

## Supporting Information

File S1 Figure S1, Sequence-specific binding activity of Nurr1 to the human TH NBREs in F3 cells. EMSA with ^32^P-labeled oligonucleotides containing the three human NBREs was performed using nuclear extracts from HB1.F3 cells transiently transfected with the pLPC-Nurr1 plasmid. Each radiolabeled NBRE oligonucleotide was incubated in the presence or absence of 40- or 80-fold molar excess of competitor DNA as indicated above the lanes. The arrowhead designates the slow-migrating complex, showing a similar pattern as in SH-SY5Y cells. Figure S2, Cross-competition assay of the NBRE-B and –C sites using EMSA. Competitions were performed with unlabeled NBRE-A, -B, and -C oligonucleotides at 40- and 80-fold excess for cross-competition with labeled NBRE-B and -C probes. Nuclear protein extract was obtained from SH-SY5Y cells transiently transfected with Nurr1-expressing plasmid. Lane 2: no transfected control; Lanes 1, 3–8: cells transfected with Nurr1-expressing plasmid; lanes 3–4: unlabeled competitor NBRE A; lanes 5–6: unlabeled competitor NBRE-B; and lanes 7–8: unlabeled competitor NBRE C. Figure S3, NBRE-B and -C mutant competition assays of Nurr1 binding. EMSA was performed with probe B or mutated probe M1 and M2. The mutations were introduced into the fourth and fifth nucleotides (M1; GG to CA) and the second and fourth nucleotides (M2; A, G to T, C) of the NBRE-B sequence in probe NBRE-B or NBRE-C. 80-fold molar excess of unlabeled oligonucleotide was added as a competitor in the reaction mixture. The retarded complex is indicated by the arrowhead. Figure S4, Recruitment of Nurr1 and SIRT1 to hTH NBRE-A site. The binding of SIRT1 and Nurr1 to hTH promoter was assayed by ChIP assays in HB1.F3 and SH-SY5Y cells. This is a representative experiment of three.
(TIF)
