# Correction: Nurr1 Represses Tyrosine Hydroxylase Expression via SIRT1 in Human Neural Stem Cells

**DOI:** 10.1371/journal.pone.0113335

**Published:** 2014-11-11

**Authors:** The PLOS ONE Staff

The second author's name is spelled incorrectly. The correct name is: Ji-Seon Seo. The correct citation is: Kim TE, Seo JS, Yang JW, Kim MW, Kausar R, et al. (2013) Nurr1 Represses Tyrosine Hydroxylase Expression via SIRT1 in Human Neural Stem Cells. PLoS ONE 8(8): e71469. doi:10.1371/journal.pone.0071469
