# Supplementary figures and images for: Nurr1 Represses Tyrosine Hydroxylase Expression via SIRT1 in Human Neural Stem Cells
Source: PLoS One. 2013 Aug 14;8(8):e71469. doi: 10.1371/journal.pone.0071469 (PMC3743743; doi:10.1371/journal.pone.0071469)

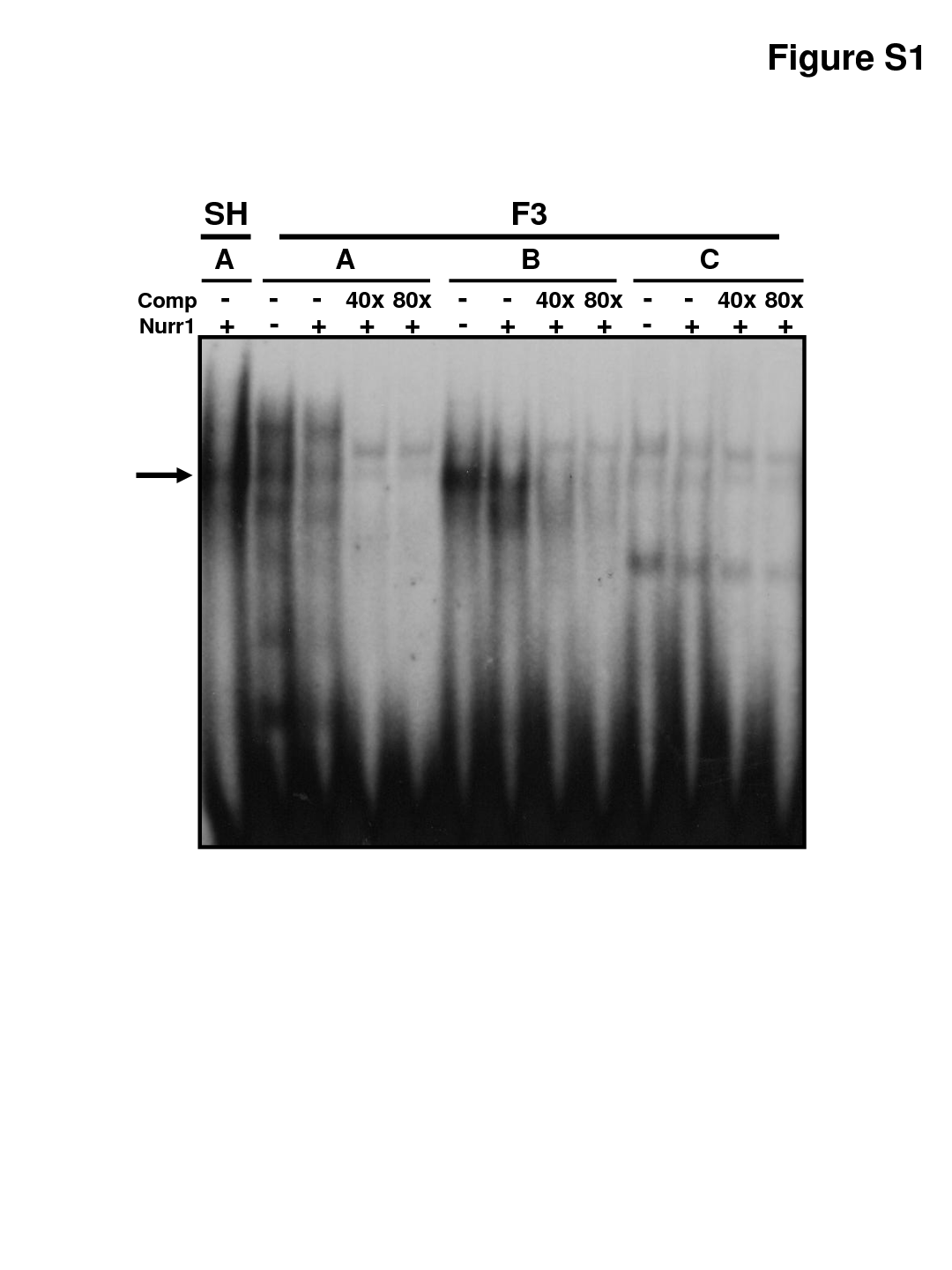

Supplement: File S1 — Figure S1, Sequence-specific binding activity of Nurr1 to the human TH NBREs in F3 cells. EMSA with 32P-labeled oligonucleotides containing the three human NBREs was performed using nuclear extracts from HB1.F3 cells transiently transfected with the pLPC-Nurr1 plasmid. Each radiolabeled NBRE oligonucleotide was incubated in the presence or absence of 40- or 80-fold molar excess of competitor DNA as indicated above the lanes. The arrowhead designates the slow-migrating complex, showing a similar pattern as in SH-SY5Y cells. Figure S2, Cross-competition assay of the NBRE-B and –C sites using EMSA. Competitions were performed with unlabeled NBRE-A, -B, and -C oligonucleotides at 40- and 80-fold excess for cross-competition with labeled NBRE-B and -C probes. Nuclear protein extract was obtained from SH-SY5Y cells transiently transfected with Nurr1-expressing plasmid. Lane 2: no transfected control; Lanes 1, 3–8: cells transfected with Nurr1-expressing plasmid; lanes 3–4: unlabeled competitor NBRE A; lanes 5–6: unlabeled competitor NBRE-B; and lanes 7–8: unlabeled competitor NBRE C. Figure S3, NBRE-B and -C mutant competition assays of Nurr1 binding. EMSA was performed with probe B or mutated probe M1 and M2. The mutations were introduced into the fourth and fifth nucleotides (M1; GG to CA) and the second and fourth nucleotides (M2; A, G to T, C) of the NBRE-B sequence in probe NBRE-B or NBRE-C. 80-fold molar excess of unlabeled oligonucleotide was added as a competitor in the reaction mixture. The retarded complex is indicated by the arrowhead. Figure S4, Recruitment of Nurr1 and SIRT1 to hTH NBRE-A site. The binding of SIRT1 and Nurr1 to hTH promoter was assayed by ChIP assays in HB1.F3 and SH-SY5Y cells. This is a representative experiment of three. (TIF) [file pone.0071469.s001.tif]
